# Prognosis of Single Early-Stage Hepatocellular Carcinoma (HCC) with CEUS Inconclusive Imaging (LI-RADS LR-3 and LR-4) Is No Better than Typical HCC (LR-5)

**DOI:** 10.3390/cancers14020336

**Published:** 2022-01-11

**Authors:** Eleonora Terzi, Alice Giamperoli, Massimo Iavarone, Simona Leoni, Ludovico De Bonis, Alessandro Granito, Antonella Forgione, Francesco Tovoli, Fabio Piscaglia

**Affiliations:** 1 Division of Internal Medicine, Hepatobiliary and Immunoallergic Diseases, IRCSS Azienda Ospedaliero Universitaria di Bologna, 40138 Bologna, Italy; eleonora.terzi@aosp.bo.it (E.T.); simona.leoni@studio.unibo.it (S.L.); alessandro.granito@unibo.it (A.G.); francesco.tovoli2@unibo.it (F.T.); 2 Department of Medical and Surgical Sciences, University of Bologna, 40138 Bologna, Italy; alice.giamperoli@studio.unibo.it (A.G.); ludovico.debonis@unibo.it (L.D.B.); antonella.forgione@studio.unibo.it (A.F.); 3 A.M. & A. Migliavacca Center for Liver Disease, Division of Gastroenterology and Hepatology, Fondazione IRCCS Ca’ Grande Maggiore Hospital, University of Milan, 20122 Milan, Italy; massimo.iavarone@policlinico.mi.it

**Keywords:** HCC, CEUS, LI-RADS, prognosis, overall survival, recurrence-free survival, imaging

## Abstract

**Simple Summary:**

The European (EASL) and American (AASLD) guidelines for the management of hepatocellular carcinoma (HCC) suggest different management of nodules with indeterminate imaging in cirrhosis. In particular, nodules classified as LR-3 and LR-4 by the CEUS LI-RADS algorithm (indeterminate for HCC) are suggested to be biopsied according the EASL guidelines, but may be only monitored with follow-up imaging, with biopsy left to selected LR-4/LR-M cases, according to the AASLD ones. The present study shows that CEUS LI-RADS classes LR-3 and LR-4 HCC have no better clinical outcome than typical HCC (LR-5). Such data support the EASL policy, aimed at conclusive diagnostic investigations of indeterminate nodules up to obtaining histological proof to avoid leaving aggressive HCC not timely treated.

**Abstract:**

The American College of Radiology (ACR) released the Liver Imaging Report and Data System (LI-RADS) scheme, which categorizes hepatic nodules in risk classes from LR-1 to LR-5 (according to the degree of risk to be HCC) and LR-M (probable malignancy not specific for HCC). The aim of this study was to test whether HCC with different LR patterns on CEUS have different overall survival (OS) and recurrence-free survival (RFS). We retrospectively enrolled 167 patients with the first definitive diagnosis of single HCC (by using CT/MRI or histological techniques if CT/MRI were inconclusive) for whom CEUS examination was available. The median size of HCC lesions was 2.2 cm (range 1.0–7.2 cm). According to CEUS LI-RADS classification, 28 patients were in LR-3, 48 in LR-4, 83 in LR-5, and 8 in LR-M. Patient liver function and nodule characteristics were not statistically different between CEUS LI-RADS classes. Using univariate analysis, CEUS LI-RADS class was not found to be a predictor of survival (*p* = 0.347). In conclusion, HCC showing the CEUS LI-RADS classes LR-3 and LR-4 have no better clinical outcome than typical HCC. Such data support the EASL policy, aimed at conclusive diagnostic investigations of indeterminate nodules up to obtaining histological proof to avoid leaving aggressive HCC not timely treated.

## 1. Introduction

The American College of Radiology (ACR)—Liver Imaging Reporting and Data System (LI-RADS) [1] is an algorithm that categorizes patients with liver nodules at risk for hepatocellular carcinoma (HCC) in different classes according to the degree of risk of nodules to be HCC and was initially designed for CT or MRI characteristics. It subgroups patients in different classes of risk of having HCC and namely LR-1 (definitely benign), LR-2 (probably benign), LR-3 (intermediate probability for HCC), LR-4 (probably HCC), LR-5 (definitely HCC), and LR-M (probable malignancy not specific for HCC). Such categories correspond to an estimated prevalence of HCC of 8–22% for LR-2, 31–45% for LR-3, 67–80% for LR-4, 92–96% for LR-5, and for LR-M 87–97% of malignancy of which 26–48% HCC [2]. Contrast-enhanced ultrasonography (CEUS) LI-RADS is a similar algorithm, but based on CEUS characteristics of liver nodules at risk for HCC, which was more recently released by the ACR [3]. The CEUS algorithm provides similar risks of HCC and malignancy for each category as that for CT/MRI [4].

The latest released AASLD guidelines [5] recommend categorization of liver nodules according to the CT/MRI LI-RADS scheme. When a definitive diagnosis of HCC (=LR-5) is not reached, the scheme recommends repeat/alternative diagnostic imaging in 3–6 months in the instance of LR-3; multidisciplinary discussion with possible repeat/alternative diagnostic imaging ≤ 3 months in the instance of LR-4/LR-M and biopsy left to selected LR-4/LR-M cases. On the opposite, the recent published EASL guidelines [6] reintroduced CEUS in the diagnostic flow-chart of liver nodules (in particular in case of inconclusive or contraindicated CT/MRI), and recommend biopsies for all the nodules without the HCC imaging hallmarks.

The aim of this study was to test whether HCC showing the CEUS pattern of indeterminate nodules (LR-3 and LR-4), typical patterns (LR-5), or LR-M patterns have different prognoses in terms of overall survival (OS) and recurrence-free survival (RFS) in order to justify or not a different tailored workup.

## 2. Results

### 2.1. Patient Characteristics

A total of 167 patients fulfilled the inclusion/exclusion criteria and made the study population. Their characteristics are reported in Table 1. The median size of nodules was 2.2 cm (range 1.0–7.2 cm). CT was performed in 53 patients (32%), MRI in 24 (14%), and both CT and MRI in 89 (53%). HCC was histologically confirmed in 97 patients (58%, most of them submitted to biopsy for inconclusive diagnosis at CT/MRI while the others had histology following hepatic resection). One patient had histological proof of HCC after CEUS had showed a pattern not typical for HCC (LR-4), but he did not receive CT/MRI imaging in our center, and radiological images and reports could not be retrieved, so he was included with CEUS only. All 167 patients were submitted to treatment and 103. HCC relapsed after a median of 10 months.

### 2.2. CEUS LI-RADS

The CEUS dynamic patterns of the HCC are reported in Table 2. The LR-3 pattern was observed in 28 patients (17%), LR-4 in 48 (29%), LR-5 in 83 (50%), and LR-M in 8 (5%). Patients and HCC characteristics subgrouped according to LI-RADS classes are reported in Table 3. CEUS LI-RADS 3 patients were significantly older than LR-4/LR-5 patients (median age 72 vs. 64 and 70 years in LR-3 vs. LR-4 and LR-5, respectively; *p* = 0.023) and LR-3 nodules smaller than the others (median diameter 16 vs. 23, 26, and 26 mm in LR-3 vs. LR-4, LR-5, and LR-M, respectively; *p* = 0.005). Furthermore, treatment techniques had a trend towards significance in different CEUS LI-RADS groups with LR-3 HCC more frequently submitted to TACE (or percutaneous treatment) than the HCC in the other LR groups (*p* = 0.107).

### 2.3. CT/MRI LI-RADS

CT and MRI LI-RADS was available only in patients from the Bologna database (97 patients out of 98 since one patient had histological proof of HCC after CEUS). CT LR-3 LI-RADS pattern was observed in 5 patients (7%), LR-4 in 6 (8%), LR-5 in 58 (80%), and LR-M in 4 (5%). MRI LR-3 LI-RADS pattern was observed in 2 patients (4%), LR-4 in 8 (15%), LR-5 in 40 (74%), and LR-M in 4 (7%).

### 2.4. Assessment of Treatment Efficacy and Relapse

HCC was treated after a median time from CEUS of 1 months (range 1–9 months). A CR at one month after a first treatment was obtained in 120/167 (72%) patients, a PR in 37 (22%), and PD in 10 (6%). Forty-one out of 47 patients with PR/PD were further submitted to sequential treatment within 1 month with bringing the total CR rate to 89% (149/167) (Table 1). Recurrence occurred in 103 patients of 149 patients achieving a CR (69%) after a median time of 10 months (range 1–113). No recurrence was documented in patients submitted to liver transplant after a median follow-up of 120 months (range 38–186 months). A local recurrence was documented in 15/103 (15%) patients, a distant intrahepatic recurrence in 62/103 (60%) patients, a distant extrahepatic recurrence in 3/103 (3%) patients, and local recurrence/distant intrahepatic recurrence in 23/103 (22%) (Table 1).

### 2.5. Overall Survival and Recurrence Free Survival

Of the total 167 HCC patients, 111 (66%) died within the study period (January 2005 to December 2020). The median overall survival for the whole study population was 49 months (range 7–186 months). At univariate survival analysis, total bilirubin ≥ 0.8 g/dL, albumin < 4 g/dL, AFP > 7.3 ng/mL, MELD score > 8, BCLC stage A, time to treatment ≤ 1 month, incomplete response after treatment, recurrence after CR and TTR ≤ 10 months were all statistically associated with shorter survival (Table 4). Interestingly, CEUS LI-RADS classes had no statistical impact on overall survival (Table 4) or recurrence free survival (RFS) (Table 5). It is worth to mentioning that the cause of death was found to have no statistical impact on survival, or the presence of mild/more relevant comorbidities (graded as Charlson index ≤ 5 and >5, respectively). Using multivariate analysis (including all variables with *p* ≤ 0.05, except for recurrence after CR and time to recurrence, see “Statistical analysis” section), time to treatment ≤ 1 month (*p* = 0.019), AFP > 7.3 ng/mL (*p* = 0.041) and incomplete response after treatment (*p* ≤ 0.001) remained as significant predictors.

Since CEUS and CT/MRI did not produce superimposable LR classes for each patient, we tested whether only CEUS produced non-statistically different survival in different LR classes, or whether CT or MRI did in addition. Results showed that even when HCCs were categorized as LR-3, LR-4, or LR-M classes by either MRI or CT (which was the cases in smaller number of cases than with CEUS), the survival outcome did not differ from that of CT/MRI LR-5, similarly to the findings obtained with CEUS (Table 4).

### 2.6. CEUS, CT, and MRI LI-RADS Classification Agreement

Agreement for LI-RADS categories between CT and MRI was moderate (κ = 0.542; 95% CI, 0.224–0.859), *p* < 0.001 (Table 6, panel A). Agreement for LI-RADS categories between CEUS and CT was slight (κ = 0.182; 95% CI, 0.00–0.283), *p* = 0.015 (Table 6, panel B). LI-RADS categories between CEUS and MRI were poorly concordant (κ = −0.034), *p* = 0.670 (Table 6, panel C) mainly related to the several LR-5 lesions at MRI showing a LR-4 pattern at CEUS, due to the lack of washout.

## 3. Discussion

In our cohort of HCC patients, 28 had CEUS LI-RADS LR-3 pattern (17%), 48 LR-4 (29%), 83 LR-5 (50%), and 8 LR-M (5%).

The present study shows that HCC nodules with different CEUS LI-RADS patterns have no different prognosis in terms of overall survival corresponding to no better biological behavior of HCC with indeterminate pattern (LR-3 and LR-4) than HCC with typical radiologic pattern (LR-5). Those results were also confirmed for the CT/MRI LI-RADS classes (Table 4), further strengthening the evidence that, although indeterminate nodules and LR-M have lower probability of being HCC than LR-5 ones [4], they have the same prognosis of LR-5 when they correspond to HCC. Noteworthy, the probability of a focal liver lesion (FLL) of being HCC in CEUS LI-RADS classes LR-3 and LR-4 is significant (47% and 86%, respectively) [4] with no LR-2-3-4 class able to rule out HCC. Similar rates have also been shown for CT and MRI [2]. These results are extremely important to guide appropriate management of indeterminate nodules in high-risk patients, suggesting prompt completion of the diagnostic work-up with the aim to timely detect tumors at an early stage and reduce the potential risk of HCC underdiagnosis and progression. This policy has been adopted by the 2018 EASL guidelines [6] that recommend biopsy for indeterminate nodules (LR-3 or LR-4) and LR-M. On the contrary, the 2018 AASLD guidelines [5] recommend different management of liver nodules according to CT/MRI LI-RADS class, suggesting repeat or alternative diagnostic imaging in 3–6 months for LR-3, multidisciplinary discussion with possible repeat/alternative diagnostic imaging ≤ 3 months in the instance of LR-4/LR-M and biopsy left to selected LR-4/LR-M cases. Importantly, also in the instance of cholangiocarcinoma, which is held as a more aggressive neoplasm than HCC, CT, or MRI imaging may be inconclusive not only for HCC but also for malignancy (differently from CEUS) [7,8,9,10,11] creating a risk of delaying diagnosis if biopsy is not promptly performed.

Biopsy of focal liver lesions is known to bear some risks (in particular bleeding and tumor seeding) that should be taken into account. Furthermore, it might suffer low sensitivity for cancer in case of smaller nodules (<2 cm). It could be also argued that the risks of invasive procedures for the diagnostic work-up of hepatic nodules could exceed the benefits of early diagnosis and treatment. However, as already stated, our data clearly suggest that HCC with a CEUS LR-3 and LR-4 pattern should not be considered less aggressive than typical (LR-5) HCC. Therefore, given the high prevalence of HCC in indeterminate nodules, the diagnostic work-up should be a priority, since it is expected to bear a positive balance between benefits and harms. This more aggressive policy after inconclusive CEUS was already proposed by Forner et al., who reported on the need of diagnostic work-up and treatment in liver nodules with different contrast profiles on CEUS [12].

It is worth pointing out that almost every nodule in the context of cirrhosis is investigated either with CT/MRI and with CEUS in our center and a thorough diagnostic work-up has always been in place in keeping with the EASL guidelines, including liver biopsy in case of pattern not typical for HCC (whenever feasible and for nodules >1 cm) [6,13,14].

A possible criticism to our CEUS results could be the fact that HCC diagnosis is not established on the CEUS pattern in first instance according to the guidelines [6] and that an indeterminate nodule at CEUS (LR-3 or LR-4) might rather show a typical HCC pattern at CT/MRI not needing further investigations. As we were very well aware of this possibility and that CEUS patterns may easily not superimpose with the CT and MRI ones, we analyzed the correlation of LI-RADS categories across different imaging modalities (CEUS, CT, and MRI) with survival outcomes. We found a slight agreement between CT and CEUS and poor agreement between MRI and CEUS (Table 6). Nonetheless, when analyzing the differences in LR categories between CEUS and MRI (Table 6) we found that when a LR-M feature was reported (either by CEUS or CT/MRI), the other imaging techniques confirmed a malignant feature namely, either LR-M or LR-5 (with only one exception for CEUS). The LR category was upgraded/downgraded by CEUS by 1 category in 26/31 patients (84%). In particular, this was the case for a number of CEUS LR-4 (which has high probability of being HCC) cases which resulted in an LR-5 category at CT/MRI, specifically in 19/31 patients (61%). On the contrary, the agreement between CT and MRI was found to be moderate as already reported [15]. Since the FLL included in the present study are all HCC, either CEUS and MRI and CT have some false negative results. The sensitivity for malignant features (LR-5 or LR-M) was higher for CT and MRI (85% and 81%, respectively) than for CEUS (60%) which was anyway not negligible and in keeping with previous reports [16,17]. Worth to mention that in the present database, CEUS LR-3 patients were statistically elderly and had smaller nodules in comparison to the other LI-RADS classes.

Concerning tumor allocation, CEUS LR-3 lesions, despite being smaller than LR-4 or LR-5, were more often submitted to TACE compared to LR-5 (50% vs. 34%), much less to surgery (4% vs. 28%) and similarly to percutaneous ablation (46% vs. 29%). This probably points out the hypothesis that LR-3 lesions had possibly resulted in LR-3 (no arterial hyperenhancement) since they were located in difficult positions, which not only prevented the best ultrasound assessment (making CEUS less performant than CT and MRI in detecting the typical LR-5 pattern), but also hampered the best treatments. The suboptimal suitability of LR-3 patients to the most curative treatment modalities (probably for tumor location and general health status of the patients) could likely justify the shorter progression free survival and overall survival, despite non statistically significant. Conversely CT and MRI do less suffer problems of explorability compared to CEUS, thus identifying LR-3 cases which are purely based on the vascular pattern. In addition, for CT and MRI no significant difference in overall survival was found in connection with the different LR classes.

Interestingly, at the univariate analysis, a delayed time to treatment (time between CEUS and HCC treatment > 1 month) was shown to be statistically associated with shorter survival, further stressing the importance of a promptly diagnostic management of indeterminate nodules in order to guarantee a timely treatment. It is worth mentioning that no impact on survival was found between patients’ death for liver failure, HCC progression or non-hepatic causes, and between patients with mild/more relevant comorbidities.

Our study has limitations. In particular, given the retrospective nature of the study, LI-RADS class was assigned on the basis of CEUS and CT/MRI original report or, whenever this lacked, on the basis of videoclips and images reviewed by expert investigators [1,3]. Thus, the reproducibility between different operators could not be tested. Nonetheless, other studies reported a good interclass correlation for final LI-RADS [4,18,19] and the vast majority of lesions in the present study showed objective and reproducible features with the reports/images reviewed by expert operators who matched opinions (reflecting the daily clinical practice). Additionally, we already verified the interobserver reproducibility in our previous study showing good results [4]. Lastly, when considering the patients included in the study, some nodules with inconclusive diagnosis for whom biopsy was deemed unfeasible or not necessary (<1 cm) were excluded from the study. It is worth pointing out that those nodules were excluded randomly mainly because not submitted to biopsy for technical reasons and not for more/less aggressive features of the nodules.

## 4. Patients and Methods

The study protocol for this retrospective study was first approved by the Ethical Committee of the Bologna S.Orsola-Malpighi Hospital (78/2017/O/OSSN). Given the retrospective design of the study and the number of patients, the acquisition of all individual informed consents was waived.

### 4.1. Patients

A total of 167 cirrhotic patients with single liver nodule referred to our Centre (n. 98 patients) between January 2005 and December 2016 and to Milan Center (n. 69) between January 2010 and March 2012, were included in the final study population. All those patients were investigated with CEUS with sulfur hexafluoride (SonoVue^®^, Bracco, Milan Italy) as a routine standard practice for all distinct newly detected nodules at risk for HCC. Analysis of the follow-up was closed in December 2020. Inclusion criteria were: (1) single HCC after staging procedures (otherwise different HCC might have had different patterns and the one impacting most on prognosis could not be ascertained); (2) presence of cirrhosis as identified risk for HCC according to the EASL guidelines [6]; (3) visible nodule investigated by CEUS; (4) availability of CEUS information reporting the arterial phase pattern, the timing of onset and the degree of washout whenever this feature occurred (either reported on the original report or, whenever this lacked, as assessed retrospectively by the investigator on recorded videoclips and images); (5) availability of an accepted diagnostic reference standard, either CT/MR scan or histology; (6) diagnosis performed within 12 weeks from the index CEUS; (7) no vascular invasion; and (8) no previously treated HCC. 

The diagnostic reference was histology, whenever available (either obtained by percutaneous biopsy or after surgical resection), or CT/MRI diagnosis of HCC according to the accepted guidelines in Italy [13,20]. Nodules without histology and not fulfilling the CT/MRI diagnostic criteria for HCC or the MRI diagnostic criteria for hemangioma were simply followed-up over time because biopsy was deemed unfeasible or not necessary (nodules < 10 mm). All those who were considered unsuitable for the aims of this study and were not included.

Disease-specific causes of death (whether related to liver failure, HCC progression or non-hepatic causes) were also collected for the entire group of patients. Furthermore, the Charlson comorbidity index was calculated in Bologna group of patients in order to quantify the clinical impact of the comorbidities, defining patients with more relevant comorbidities for a Charlson index > 5.

### 4.2. CEUS Pattern and LI-RADS Classification

The combination of the arterial and venous phase appearances allowed classification of nodules as LR-3, LR-4, LR-5, and LR-M classes according to the LI-RADS scheme [1,21]. Arterial phase hyperenhancement was defined as a lesion becoming globally or partially hyperenhanced (but not with rim or globular peripheral distribution) compared to the surrounding parenchyma in the arterial phase. “Washout” is defined when the lesion became hypoenhanced compared to the surrounding parenchyma in the portal-venous phase [1,10]. When such a washout occurred, it was further classified according to its timing as “early” if it appeared before 60 s following contrast injection or as “late” if occurring later, and to its intensity as “marked” when the lesion became markedly hypoenhanced or punched out (otherwise defined as “mild”) within 2 min [21,22]. A rim enhancement pattern (not globular peripheral) in the arterial phase categorized the lesion as LR-M, regardless of the venous pattern. Further, marked and/or early onset venous washout classified a lesion as LR-M regardless of the arterial appearance. LR-5 class comprised nodules ≥ 10 mm with arterial phase hyperenhancement (either global or in part) followed by washout appearance that was mild in degree and late in onset [1,3,21,22]. The LR-3 category comprised nodules with iso- or hypoenhancement in the arterial phase without later washout regardless of their size or, in the instance of the occurrence of mild and late washout only if <20 mm in size. If the latter pattern occurred in a lesion ≥ 20 mm in size this was coded as LR-4. LR-4 category also included lesions ≥ 10 mm with arterial phase hyperenhancement (excluded rim and globular peripheral), but without any washout [1].

The original report of CEUS was reviewed (and LI-RADS class assigned) and, whenever this lacked or in case of doubtful report, we recovered the clips (mostly) and/or still relevant images of the needed vascular contrast phases and categorized them according to the LI-RADS CEUS pattern.

### 4.3. CT/MRI LI-RADS Classification

An abdominal CT and/or MRI was available in 166 patients from the entire database. The original report of CT/MRI was reviewed only for the 98 patients from Bologna (and LI-RADS class assigned) and, whenever this lacked, a retrospective assessment of images by expert radiologists was performed. There was no prompt digital archival system in Milan to be utilized.

### 4.4. Treatment and Assessment of Treatment Efficacy

A treatment was delivered in all 167 patients with single HCC and the time to treatment (time between CEUS and HCC treatment) calculated.

Post-treatment radiological assessment of tumor response was performed by CT or MRI in all patients, at approximately 1 month. In all 167 patients with single HCC of locoregional treatment and at 2–3 months in case of surgical resection. Follow-up imaging (at 3–4 months intervals) were then performed in all cases with complete response in order to assess the appearance of recurrence. The response was considered complete (CR) when a disappearance of any intratumoral arterial enhancement was documented after locoregional therapies and when the tumoral lesion had disappeared in case of surgical resection [22].

Transplanted patients were submitted to close ultrasound (US) follow-up at 1 and 3 months and every 6 months thereafter in order to detect recurrence. A CT was performed every 6 months for the first 2 years and then with dilated time intervals on the basis of US findings. Time to recurrence was calculated as the time interval between the time of documented complete response and any possible HCC recurrence, either local or distant.

### 4.5. Statistical Analysis

Continuous variables were reported with their median and range. Descriptive analysis was carried out reporting rates in absolute numbers and percentages. Comparisons among groups were calculated using non-parametric tests (Mann–Whitney and Wilcoxon). Categorical variables were compared with the χ^2^ test. All tests were considered significant at *p* < 0.05.

OS was defined as the time interval between HCC diagnosis and death or the date of the last follow-up information about being dead or alive; RFS was defined as the time interval between HCC treatment with complete response and recurrence, death or the date of the last follow-up information about tumor status. Survival curves were computed according to Kaplan–Meier methods and compared by log-rank tests. Variables with *p* < 0.05 in univariate analysis were entered into a stepwise Cox regression model (conditional backward selection), except for recurrence after CR and time to recurrence in order to avoid a selection bias since those include only a subgroup of patients. For patients who stopped attending the facilities of our hospital, information on the living status or time of death were retrieved by enquiries to the registry offices of the patients’ hometowns.

Agreement for LI-RADS categories among CEUS, CT, and MRI was assessed using κ statistics. Agreement was interpreted based on Cohen’s kappa intraclass coefficient (κ) as follows: 0.20 or less, slight agreement; 0.21 to 0.40, fair agreement; 0.41 to 0.60, moderate agreement; 0.61 to 0.80, substantial agreement; and 0.81 to 1.00, almost perfect agreement.

Analysis of the data was performed using SPSS version 25.0.

## 5. Conclusions

In conclusion, HCC showing the CEUS (or CT/MRI) LI-RADS classes LR-3 and LR-4 held inconclusive for malignancy have no better clinical outcome than typical LR-5 HCC. Therefore, completion of diagnostic workup including the integration of other imaging techniques up to biopsy should be promptly pursued to avoid leaving aggressive HCC undiagnosed and consequently not timely treated.

## Figures and Tables

**Table 1:** Patients and HCC characteristics.

Variables	Patients/HCC (*n* = 167)
**Gender male, *n* (%)**	88 (53)
**Age, median (years) (range)**	69 (40–88)
**Etiology of cirrhosis, *n* (%)**	
- HCV - HBV - Alcohol - NASH - Others	114 (68) 17 (10) 13 (8) 11 (7) 12 (7)
Presence of ascites *, *n* (%)	15 (9)
**Presence of hepatic encephalopathy, *n* (%)**	1 (1)
**Presence of portal hypertension, *n* (%)**	86 (52)
**INR, median (range)**	1.14 (0.9–2.2)
**Total bilirubin, median (mg/dL) (range)**	0.8 (0.3–2.9)
**Albumin, median (g/dL) (range)**	4.0 (2.8–5.5)
**Creatinine, median (mg/dL) (range)**	0.9 (0.4–1.8)
**AFP, median (ng/mL) (range)**	7.3 (1–8446)
**BCLC stage, *n* (%)**	
- 0 - A	34 (20) 133 (80)
**MELD score median (range)**	8 (6–18)
**Tumor size, median (mm) (range)**	22 (10–72)
**Tumor size > 50 mm, *n* (%)**	9 (5)
**Histological diagnosis, *n* (%)**	97 (58)
**Tumor grade sec. Edmondson, *n* (%)**	
- 1–2 - 3–4	66 (68) 31 (32)
**Available CT imaging, *n* (%)**	53 (32)
**Available MRI imaging, *n* (%)**	24 (14)
**Available CT and MRI imaging, *n* (%)**	89 (53)
**CEUS arterial-phase hyperenhahcement, *n* (%)**	138 (83)
**CEUS washout on portal venous phase, *n* (%)**	92 (55)
**CEUS LI-RADS classes, *n* (%)**	
- LR-3 - LR-4 - LR-5 - LR-M	28 (17) 48 (28) 83 (50) 8 (5)
**HCC treatment, *n* (%)**	
- TACE - Percutaneous ablation - Resection	67 (40) 67 (40) 33 (20)
**Time to treatment, median (months) (range)**	1 (1–9)
**Tumor response after first treatment, *n* (%)**	
- Complete response (CR) - Partial response (PR) - Progressive disease (PD)	120 (72) 37 (22) 10 (6)
Total CR achievement after other sequential therapies, *n* (%) *^2^	149 (89)
**HCC recurrence after CR, *n* (%)**	103 (69)
**TTR, median (months) (range)**	10 (1–113)
**Transplant, *n* (%)**	22 (13)
**Type of recurrence, *n* (%)**	
- Local recurrence - Distant intrahepatic recurrence - Distant extrahepatic recurrence - Local recurrence + distant intrahepatic recurrence	15 (15) 62 (60) 3 (3) 23 (22)
**Death, *n* (%)**	111 (66)
**Cause of death, *n* (%)**	
- Non liver-related - Liver failure - Tumor progression	25 (23) 13 (12) 73 (65)
Charlson index, *n* (%) *^1^	
- ≤5 - >5 - >5	26 (27) 72 (73)

* Not clinically detectable, only at imaging. *^1^ Available only in patients from the Bologna database. *^2^ Besides first treatment.

**Table 2:** CEUS LI-RADS subcategories and relative frequencies.

	Arterial Phase HYPO/Isoenhancement		Arterial Phase Hyperenhancement ^1^	
**Nodule Size (mm)**	<20	≥20	<10	≥10
**No Washout of Any Type**	CEUS LR-3 16 HCC (10%)	CEUS LR-3 11 HCC (7%)	CEUS LR-3 0 HCC (0%)	CEUS LR-4 47 HCC (28%)
**Late and Mild Washout ^2^**	CEUS LR-3 1 HCC (1%)	CEUS LR-4 1 HCC (1%)	CEUS LR-4 0 HCC (0%)	CEUS LR-5 83 HCC (50%)

^1^ Arterial phase hyperenhancement whole or in part did not rim peripheral discontinuous globular enhancement; ^2^ late in onset (60 s) and mild in degree: in whole or in part with no part showing early or marked washout.

**Table 3:** Patients and tumor characteristics in different CEUS LI-RADS categories.

Total Patients/HCC = 167 *n* (%)	Total	LR-3 28 (17)	LR-4 48 (29)	LR-5 83 (50)	LR-M 8 (5)	*p*
Patient’s gender male, *n* (%)	88 (53)	12 (43)	27 (56)	44 (53)	5(62)	0.650
Patient’s age, median (years) (range)	69 (40–88)	72 (40–85)	64 (44–82)	70 (46–88)	73 (49–85)	**0.023**
Etiology HCV, *n* (%)	114 (68)	21 (75)	31 (65)	5 (66)	7 (87)	0.493
Presence of ascites, *n* (%)	15 (9)	4 (14)	4 (8)	7 (8)	0 (0)	0.613
Presence of portal hypertension, *n* (%)	86 (52)	15 (57)	29 (60)	38 (46)	4 (50)	0.445
INR, median (range)	1.14 (0.9–2.2)	1.1 (0.9–1.6)	1.2 (0.9–2.1)	1.1 (0.9–2.2)	1.1 (0.9–1.5)	0.477
Bilirubin, median (mg/dL) (range)	0.8 (0.3–2.9)	0.8 (0.5–2.9)	0.9 (0.3–2.7)	0.8 (0.2–2.9)	0.7 (0.5–2.6)	0.366
Albumin, median (g/dL) (range)	4.0 (2.8–5.5)	3.9 (2.9–5.2)	3.9 (2.8–5.2)	4.0 (2.8–5.4)	4.0 (3.3–4.6)	0.775
Creatinine, median (mg/dL) (range)	0.9 (0.4–1.8)	0.9 (0.7–1.5)	0.9 (0.4–1.7)	0.9 (0.5–1.6)	0.9 (0.6–1.8)	0.554
AFP, median (ng/mL) (range)	7.3 (1–8446)	11.8 (2–1924)	7 (1–3050)	7.0 (1–8446)	4 (2–50)	0.578
MELD score, median (range)	8 (6–18)	8.5 (6–18)	9 (6–17)	8 (6–18)	9.5 (6–17)	0.831
BCLC stage 0, *n* (%)	34 (20)	8 (29)	10 (21)	14 (17)	2 (5)	0.593
BCLC stage A, *n* (%)	133 (80)	20 (71)	38 (79)	69 (83)	6 (75)	0.593
Nodule size, median (mm) (range)	22 (10–72)	16 (10–31)	23 (10–51)	22 (12–72)	26 (13–55)	**0.005**
Tumor grade sec. Edmondson, *n* (%)						0.107
G1–G2	66 (68)	17 (77)	21 (78)	26 (62)	2 (33)	
G3–G4	31(32)	5 (23)	6 (22)	16 (38)	4 (67)	
First treatment, *n* (%)						0.107
TACE	67 (40)	14 (50)	23 (48)	28 (34)	2 (25)	
RF o PEI	67 (40)	13 (46)	18 (37)	32 (39)	4 (50)	
Resection	33 (20)	1 (4)	7 (15)	23 (28)	2 (25)	
Time to treatment in months, median (range)	1 (1–9)	1 (1–6)	1.5 (1–9)	1 (1–7)	1 (1–3)	0.897
CR achievement	149 (89)	27 (94)	42 (87)	73 (88)	7 (87)	0.610
Recurrence after CR, *n* (%)	103 (62)	17 (63)	30 (71)	51 (70)	5 (71)	0.892
Time to recurrence, median (range)	10 (1–113)	8 (1–26)	11 (1–62)	9.5 (2–113)	6 (1–22)	0.425
Type of recurrence, *n* (%)						0.658
Local recurrence	15 (15)	1 (6)	3 (10)	11 (21)	1 (20)	
Distant intrahepatic recurrence	62 (60)	8 (47)	21 (70)	29 (57)	3 (60)	
Distant extrahepatic recurrence	3 (3)	1 (6)	0 (0)	2 (4)	0 (0)	
Local recurrence + distant	23 (22)	7 (41)	6 (20)	9 (18)	1 (20)	
Intrahepatic recurrence						
Transplant patients, *n* (%)	22 (13)	4 (14)	7 (15)	10 (12)	1 (12)	0.976
Cause of death, *n* (%)						0.937
Non liver-related	25 (23)	4 (18)	8 (26)	12 (23)	1 (17)	
Liver failure	13 (12)	3 (14)	3 (10)	7 (13.5)	0 (0)	
Tumor progression	73 (65)	15 (68)	20 (64)	33 (63.5)	5 (83)	
Charlson index, *n* (%) *						0.680
≤5	26 (27)	2 (25)	10 (32)	12 (22)	2 (40)	
>5	72 (73)	6 (75)	21 (68)	42 (78)	3 (60)	

* Available only in patients from the Bologna database. Significant tests (*p* < 0.005) are in bold font.

**Table 4:** Univariate survival analyses.

Variable (*n*)	Survival *n*, (%)			Median Survival (95% C.I.)	*p*
	1 Year	3 Years	5 Years		
**Gender**					0.201
Male (88)	82 (93)	56 (50)	37 (47)	51.0 (27.8–74.1)	
Female (79)	96 (76)	51(64)	36 (45)	51.0 (18.4–83.5)	
**Age, years**					0.620
≤69 (86)	83 (96)	50 (58)	36 (45)	41.0 (21.8–60.1)	
>69 (81)	75 (92)	51 (63)	37 (48)	55.0. (30.1–79.9)	
**Etiology**					0.963
HCV (114)	107 (93)	70 (61)	48 (45)	44.0 (25.3–62.6)	
Others (53)	51 (96)	31 (58)	24 (47)	57.0 (27.6–86.3)	
**INR**					0.060
INR < 1.14 (87)	82 (94)	57 (65)	42 (51)	70.0 (33.5–106.4)	
INR ≥ 1.14 (80)	76 (95)	44 (55)	30 (39)	38.0 (30.83–45.6)	
**Total bilirubin, mg/dL**					**0.017**
<0.8 (80)	77 (96)	56 (70)	42 (53)	66.0 (26.1–105.8)	
≥0.8 (87)	81 (93)	45 (51)	30 (39)	38.0 (28.8–47.1)	
**Albumin, g/dL**					**0.034**
<4.0 (82)	75 (91)	46 (56)	28 (38)	30.0 (26.7–53.2)	
≥4.0 (85)	83 (97)	55 (64)	44 (54)	70.0 (28.6–111.3)	
**Creatinine, mg/mL**					0.546
<0.9 (82)	80 (97)	47 (57)	33 (44)	51.0 (29.3–72.6)	
≥0.9 (85)	78 (91)	54 (63)	40 (48)	51.0 (24.4–77.5)	
**AFP, ng/mL**					0.193
≤20 (121)	115 (95)	75 (62)	57 (49)	60.0 (34.4–85.5)	
>20 (46)	43 (93)	26 (56)	15 (35)	38.0 (30.2–45.7)	
**AFP, ng/mL**					**0.002**
≤7.3 (84)	81 (96)	56 (67)	56 (46)	82.0 (30.4–133.6)	
>7.3 (83)	78 (94)	45 (54)	26 (37)	51.0 (34.1–67.9)	
**MELD score**					**0.039**
≤8 (84)	80 (95)	56 (66)	41 (52)	66.0 (22.1–109.8)	
>8 (83)	78 (94)	45 (54)	31 (40)	39.0 (20.9–47.0)	
**BCLC score**					**0.017**
0 (34)	34 (100)	26 (76)	17 (55)	61.0 (17.8–104.2)	
A (133)	124 (93)	75 (56)	55 (44)	43.0 (27.3–58.6)	
**Nodule size, mm**					0.053
≤20 (77)	74 (96)	49 (64)	34 (49)	57.0 (00.4–113.6)	
>20 (90)	84 (93)	58 (52)	44 (38)	51.0 (8.6–67.9)	
**Tumor grade sec. Edmondson**					0.095
G1–G2 (66)	62 (94)	48 (73)	38 (59)	100.0 (47.4–152.6)	
G3–G4 (31)	29 (94)	14 (45)	9 (35)	65.0 (16.2–113.7)	
**First treatment**					0.634
TACE (67)	62 (92)	36 (53)	26 (40)	43.0 (23.5–62.4)	
RF or PEI (67)	65 (97)	44 (65)	33 (49)	51.0 (13.2–88.7)	
Resection (33)	31 (93)	21 (63)	15 (53)	65.0 (24.8–105.1)	
**Time to treatment, months**					**0.036**
≤1 (97)	91 (94)	64 (66)	49 (55)	73 (34.4–112.5)	
>1 (70)	67 (95)	37 (53)	23 (34)	38 (30.7–45.2)	
**Total CR**					**<0.001**
Yes (149)	145 (97)	98 (65)	70 (50)	61.0 (38.9–83.0)	
No (18)	13 (72)	3 (16)	2 (11)	15.0 (13.9–16.0)	
**Recurrence after CR**					**<0.001**
Yes (103)	100 (97)	61 (59)	40 (41)	44.0 (30.8–57.1)	
No (46)	45 (97)	37 (80)	33 (71)	107.5 (62.2–152.8)	
**Time to recurrence, months**					**0.001**
≤10 (54)	51 (94)	23 (42)	15 (29)	30.0 (23.8–36.1)	
>10 (49)	49 (100)	38 (77)	25 (54)	65.0 (36.6–93.3)	
**CEUS LI-RADS class**					0.347
LR-3 (28)	26 (92)	13 (46)	7 (25)	31.0 (23.2–38.7)	
LR-4 (48)	47 (97)	30 (62)	22 (47)	57.0 (22.3–91.6)	
LR-5 (83)	77 (92)	53 (63)	42 (52)	66.0 (20.4–111.5)	
LR-M (8)	8 (100)	5 (62)	4 (50)	55.0 (0.0–114.5)	
**Cause of death**					0.701
Non liver-related (25)	22 (88)	13 (52)	6 (24)	37 (24.0–49.9)	
Liver failure (13)	12 (92)	5 (38)	2 (15)	32 (15.5–48.4)	
Tumor progression (73)	68 (93)	27 (37)	14 (19)	30 (27.7–32.2)	
Charlson index *					0.194
≤5 (26)	26 (100)	14 (54)	10 (45)	41 (4.3–77.6)	
>5 (72)	67 (93)	44 (61)	27 (42)	51 (36.5–65.4)	
CT LI-RADS class *					0.821
LR-3 (5)	5 (100)	4 (80)	3 (60)	61.0 (22.3–99.6)	
LR-4 (7)	7 (100)	4 (57)	3 (42)	55.0 (0.0–119.1)	
LR-5 (57)	55 (96)	38 (66)	25 (52)	61.0 (45.2–76.7)	
LR-M (4)	4 (100)	3 (75)	2 (50)	57.0	
MRI LI-RADS class *					0.749
LR-3 (2)	2 (100)	2 (100)	0 (0)	53.0	
LR-4 (8)	7 (87)	4 (50)	4 (50)	34.0 (0.0–86.1)	
LR-5 (40)	37 (92)	20 (50)	15 (37)	36.0 (25.6–46.3)	
LR-M (4)	4 (100)	3 (75)	2 (50)	41.0	

* Available only in patients from the Bologna database. Significant tests (*p* < 0.005) are in bold font.

**Table 5:** Recurrence-free survival in different CEUS LI-RADS classes and different first treatments.

Variable	Recurrence Free (%)		Median RFS (95% C.I.)	*p*
	1 Year	3 Years		
**CEUS LI-RADS class**				0.934
LR-3	59	22	15 (8.2–21.7)	
LR-4	54	23	18 (10.2–25.7)	
LR-5	62	28	24 (12.0–35.9)	
LR-M	42	28	8 (2.8–13.1)	
**First treatment**				0.958
TACE	55	31	14 (5.6–22.3)	
RF or PEI	61	35	22 (10.0–33.9)	
Resection	59	33	22 (3.3–40.6)	

**Table 6:** Agreement for LI-RADS category assignment between: A—CT versus MRI; B—CEUS versus CT; C—CEUS versus MRI.

CT versus MRI (A)	**MRI**	**CT**			
		**LR-3**	**LR-4**	**LR-5**	**LR-M**
	LR-3	**0**	0	1	0
	LR-4	0	**2**	1	0
	LR-5	2	1	**19**	0
	LR-M	0	0	1	**3**
CEUS versus CT (B)	**CEUS**	**CT**			
		**LR-3**	**LR-4**	**LR-5**	**LR-M**
	LR-3	**0**	1	3	0
	LR-4	2	**4**	11	3
	LR-5	3	1	**40**	1
	LR-M	0	1	3	**0**
CEUS versus MRI (C)	**CEUS**	**MRI**			
		**LR-3**	**LR-4**	**LR-5**	**LR-M**
	LR-3	**1**	2	5	0
	LR-4	1	**1**	14	3
	LR-5	0	5	**18**	1
	LR-M	0	0	3	**0**

Concordant results are in bold font.

## Data Availability

The data that support the findings of this study are available from the corresponding author upon reasonable request.

## References

[1] https://www.acr.org/Quality-Safety/Resources/LIRADS.

[2] van der Pol C., Lim C.S., Sirlin C.B., McGrath T.A., Salameh J.-P., Bashir M.R., Tang A., Singal A.G., Costa A.F., Fowler K. (2019). Accuracy of the Liver Imaging Reporting and Data System in Computed Tomography and Magnetic Resonance Image Analysis of Hepatocellular Carcinoma or Overall Malignancy—A Systematic Review. Gastroenterology.

[3] Kono Y., Lyshchik A., Cosgrove D., Dietrich C.F., Jang H.J., Kim T.K., Kim T.K., Piscaglia F., Willmann J.K., Wilson S.R. (2017). Contrast Enhanced Ultrasound (CEUS) Liver Imaging Reporting and Data System (LI-RADS(R)): The official version by the American College of Radiology (ACR). Ultraschall Med..

[4] Terzi E., Iavarone M., Pompili M., Veronese L., Cabibbo G., Fraquelli M., Riccardi L., De Bonis L., Sangiovanni A., Leoni S. (2018). Contrast ultrasound LI-RADS LR-5 identifies hepatocellular carcinoma in cirrhosis in a multicenter restropective study of 1,006 nodules. J. Hepatol..

[5] Marrero J.A., Kulik L.M., Sirlin C.B., Zhu A.X., Finn R.S., Abecassis M.M., Roberts L.R., Heimbach J.K. (2018). Diagnosis, Staging, and Management of Hepatocellular Carcinoma: 2018 Practice Guidance by the American Association for the Study of Liver Diseases. Hepatology.

[6] European Association for the Study of the Liver (2018). EASL Clinical Practice Guidelines: Management of hepatocellular carcinoma. J. Hepatol..

[7] Galassi M., Iavarone M., Rossi S., Bota S., Vavassori S., Rosa L., Leoni S., Venerandi L., Marinelli S., Sangiovanni A. (2013). Patterns of appearance and risk of misdiagnosis of intrahepatic cholangiocarcinoma in cirrhosis at contrast enhanced ultrasound. Liver Int..

[8] Granito A., Galassi M., Piscaglia F., Romanini L., Lucidi V., Renzulli M., Borghi A., Grazioli L., Golfieri R., Bolondi L. (2013). Impact of gadoxetic acid (Gd-EOB-DTPA)-enhanced magnetic resonance on the non-invasive diagnosis of small hepatocellular carcinoma: A prospective study. Aliment. Pharmacol. Ther..

[9] Iavarone M., Piscaglia F., Vavassori S., Galassi M., Sangiovanni A., Venerandi L., Forzenigo L.V., Golfieri R., Bolondi L., Colombo M. (2013). Contrast enhanced CT-scan to diagnose intrahepatic cholangiocarcinoma in pa-tients with cirrhosis. J. Hepatol..

[10] Piscaglia F., Iavarone M., Galassi M., Vavassori S., Renzulli M., Forzenigo L.V., Granito A., Salvatore V., ·Sangiovanni A., Golfieri R. (2015). Cholangiocarcinoma in Cirrhosis: Value of Hepatocyte Specific Magnetic Reso-nance Imaging. Dig. Dis..

[11] Sagrini E., Iavarone M., Stefanini F., Tovoli F., Vavassori S., Maggioni M., Renzulli M., Salvatore V., Stefanescu H., Colombo M. (2019). Imaging of combined hepatocellular-cholangiocarcinoma in cirrhosis and risk of false diagnosis of hepatocellular carcinoma. United Eur. Gastroenterol. J..

[12] Forner A., Vilana R., Bianchi L., Rodríguez-Lope C., Reig M., García-Criado M.Á., Rimola J., Solé M., Ayuso C., Bru C. (2015). Lack of arterial hypervascularity at contrast-enhanced ultrasound should not define the priority for diagnostic work-up of nodules. J. Hepatol..

[13] European Association for the Study of the Liver (2012). EASL-EORTC clinical practice guidelines: Management of hepatocellular carcinoma. J. Hepatol..

[14] Bruix J., Sherman M., Llovet J.M., Beaugrand M., Lencioni R., Burroughs A.K., Christensen E., Pagliaro L., Colombo M., Rodés J. (2001). Clinical Management of Hepatocellular Carcinoma. Conclusions of the Barcelona-2000 EASL Conference. J. Hepatol..

[15] Chernyak V., Flusberg M., Law A., Kobi M., Paroder V., Rozenblit A.M. (2018). Liver Imaging Reporting and Data System: Discordance Between Computed Tomography and Gadoxetate-Enhanced Magnetic Resonance Imaging for Detection of Hepatocellular Carcinoma Major Features. J. Comput. Assist. Tomogr..

[16] SanGiovanni A., Manini M.A., Iavarone M., Romeo R., Forzenigo L.V., Fraquelli M., Massironi S., Della Corte C., Ronchi G., Rumi M. (2009). The diagnostic and economic impact of contrast imaging techniques in the diagnosis of small hepatocellular carcinoma in cirrhosis. Gut.

[17] Forner A., Vilana R., Ayuso C., Bianchi L., Solé M., Ayuso J.R., Boix L., Sala M., Varela M., Llovet J.M. (2008). Diagnosis of hepatic nodules 20 mm or smaller in cirrhosis: Prospective validation of the noninvasive diagnostic criteria for hepatocellular carcinoma. Hepatology.

[18] Fowler K.J., Tang A., Santillan C., Bhargavan-Chatfield M., Heiken J., Jha R.C., Weinreb J., Hussain H., Mitchell D.G., Bashir M.R. (2018). Interreader Reliability of LI-RADS Version 2014 Algorithm and Imaging Features for Diagnosis of Hepatocellular Carcinoma: A Large International Multireader Study. Radiology.

[19] Bolondi L., Cillo U., Colombo M., Craxì A., Farinati F., Giannini E.G., Massimo R.G., Antonio L., Pinna D., Piscaglia F. (2013). Position paper of the Italian Association for the Study of the Liver (AISF): The multi-disciplinary clinical approach to hepatocellular carcinoma. Dig. Liver Dis..

[20] Piscaglia F., Wilson S.R., Lyshchik A., Cosgrove D., Dietrich C.F., Jang H.J., Kim T.K., Salvatore V., Willmann J.K., Sirlin C.B. (2017). American College of Radiology Contrast Enhanced Ultrasound Liver Imaging Reporting and Data System (CEUS LI-RADS) for the diagnosis of Hepatocellular Carcinoma: A pictorial essay. Ultraschall Med..

[21] Piscaglia F., Kudo M., Han K.H., Sirlin C. (2017). Diagnosis of Hepatocellular Carcinoma with Non-Invasive Imaging: A Plea for Worldwide Adoption of Standard and Precise Terminology for Describing Enhancement Criteria. Ultraschall Med. Eur. J. Ultrasound.

[22] Lencioni R., Llovet J.M. (2010). Modified RECIST (mRECIST) Assessment for Hepatocellular Carcinoma. Semin. Liver Dis..

